# Immediate Effect of Hoof Trimming on Hoof and Thoracic Joint Angles in Mangalarga Mares

**DOI:** 10.3390/ani13152490

**Published:** 2023-08-02

**Authors:** Marina Lansarini Antonioli, Paulo Aléscio Canola, Júlia Ribeiro Garcia de Carvalho, Mayara Gonçalves Fonseca, Guilherme de Camargo Ferraz

**Affiliations:** 1 SCIEN—Large Animal Surgery Lab, Department of Veterinary Medicine and Surgery, School of Agricultural and Veterinary Sciences, São Paulo State University, FCAV/UNESP, Jaboticabal 14884-900, SP, Brazil; 2 Department of Animal Morphology and Physiology, School of Agricultural and Veterinary Sciences, São Paulo State University, FCAV/UNESP, Jaboticabal 14884-900, SP, Brazil

**Keywords:** equine, hoof, trimming, joint angles, photographs

## Abstract

**Simple Summary:**

Trimming is routinely performed in horses, with direct effects on hoof structures; however, the immediate effect on forelimb joints is unknown. Thus, we investigated the immediate effect of trimming on hoof structures and angulation of the thoracic limb joints through linear and angular measurements. Differences were found in hoof length, toe angle, heel angle, medial heel height, and metacarpophalangeal angle after the procedure, proving the immediate effect of the procedure. Correlations were found between trimming and the proximal joint angles.

**Abstract:**

It is important to understand the effects of hoof trimming on hoof and limb conformation to maximize its benefits on the health of the appendicular skeleton of horses, thus promoting improvements in athletic performance and sporting longevity with regard to athletic horses. There is little information on possible changes in the angulation of the thoracic limb joints after hoof trimming and correlations between the angulation of the thoracic limb joints with hoof measurements. To that purpose, nineteen Mangalarga mares received routine hoof trimming. Visual recordings (photographs) were taken before and after the procedure. Differences (*p* < 0.05) were found in hoof length, toe angle, heel angle, medial heel height, and metacarpophalangeal angle. Before trimming, correlations were found between frog length and scapulohumeral angle (SH) (*r* = −0.457; *p* = 0.049), and between toe length and shoulder-ground angle (SG) (*r* = −0.553; *p* = 0.049). A correlation was also seen between the distance from the frog to the lateral wall and the SH angle (*r* = 0.690; *p* = 0.001). After trimming, there was a correlation between humeroradial (HR) and SH joint angles (*r* = 0.669; *p* = 0.002), and the SG and SH angles (*r* = 0.488; *p* = 0.034). This study showed an immediate effect of trimming on the toe angle and heel angle and on the metacarpophalangeal joint angle, in addition to correlations between the hoof and proximal joint angles, following trimming, thus evidencing the relevance of trimming not only in hoof morphology, but also in the conformation of the appendicular skeleton of horses.

## 1. Introduction

Hoof trimming and shoeing are routine procedures with significant effects on the health of horses and may influence the performance of athletic horses. They alter the external and internal structures of the hoof, directly influencing the entire limb [1]. Good farriery should balance the hooves to facilitate optimal movement, prevent injury, and improve athletic performance [2,3]. The term ‘hoof balance’ refers not only to the geometric shape of the hoof, but also to how the hoof and each limb interact with the contact surface [4], including dorsopalmar balance. Trimming must consider the genetics, conformation, environmental influences, and athletic activities of the animal, with regard to its specific anatomical conformation [1]. 

Trimming and shoeing influence not only the morphology of the hooves [2,5,6] but also their balance [7]. In addition, correlations between hoof morphometric changes with stride length and with changes in joint angles [8,9,10,11,12] are described. Even so, there is little information relating to changes in joint angles and possible correlations with hoof measurements attributed to trimming. Most of the previous studies made evaluations after regular trimming or shoeing intervals, and considerably few studies [10] bring information about the changes found in hoof measurements immediately after trimming, thus evaluating the morphometric changes subsequent to trimming.

Since hoof trimming can impact the entire limb [1], changes in biomechanics, particularly in the forces acting on the hoof [13] and in the moments of rotation that act around the distal interphalangeal joint [14], are expected. In view of this, alterations in the distal and proximal joint angles which play a fundamental role in the animal’s movements [15], and affect the type and quality of the stride [16], should be considered. 

Accurate hoof measurements can be made from photographs and radiographs using commercially available software [17]. Likewise, joint angles can be measured using anatomical markers as reference points [8,18,19].

Changes in hoof conformation due to routine trimming and its effect on proximal and distal thoracic limb joint angles have not been previously reported in Mangalarga horses. In so, we ought to evaluate the immediate effects of trimming on hoof morphometry, and proximal and distal joint angles, as well as to investigate existing correlations between hoof measurements and joint angles before and after trimming.

## 2. Materials and Methods

The project was approved and supervised by the institution’s animal use and care committee (protocol 018789/17). Nineteen healthy Mangalarga mares, embryo donors, with a mean age of 10.37 ± 4.55 years and a mean body weight of 500.75 ± 47.07 kg, were included in this study. All animals lived under a semi-extensive regime and were distributed in two distinct farms. None of the horses performed mounted or guided work, and they were used only as embryo donors. None of the mares showed obvious lameness at pace or trot previous to the study. Regular hoof trimming was performed with an interval between six and eight weeks, and none of the mares had been shod in the previous six months. The animals were kept in paddocks, where hay and commercial feed were offered twice a day, with free access to potable water. 

**Figure 1:**
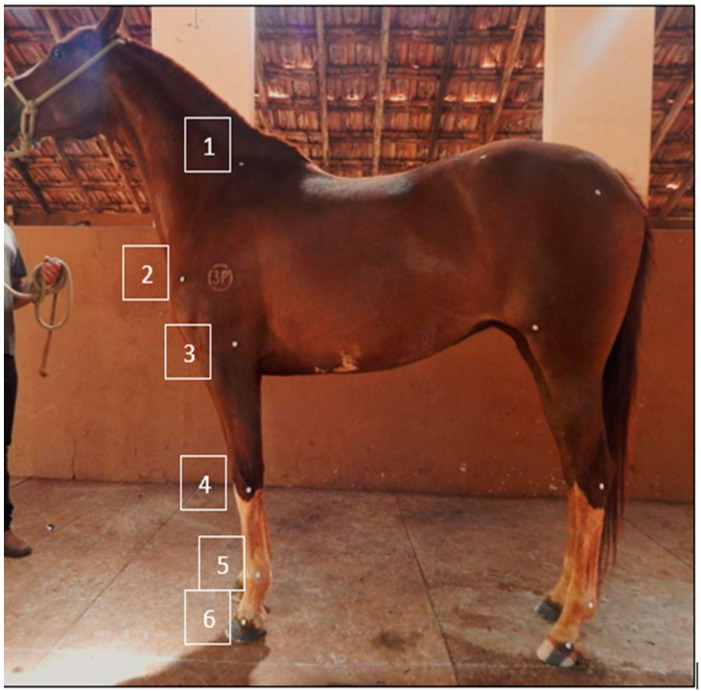
Photographic recording of a Mangalarga mare demonstrating the anatomical positioning of the reflective markers on the left thoracic limb. Regarding the numbers, 1—the superior dorsal portion of the spine of the shoulder; 2—the central area of the scapulohumeral joint; 3—radial tuberosity just below the glenoid cavity; 4—the middle third of the carpal joint; 5—the middle third of the metacarpophalangeal joint; 6—proximal interphalangeal joint of the forelimb (coronary band).

**Figure 2:**
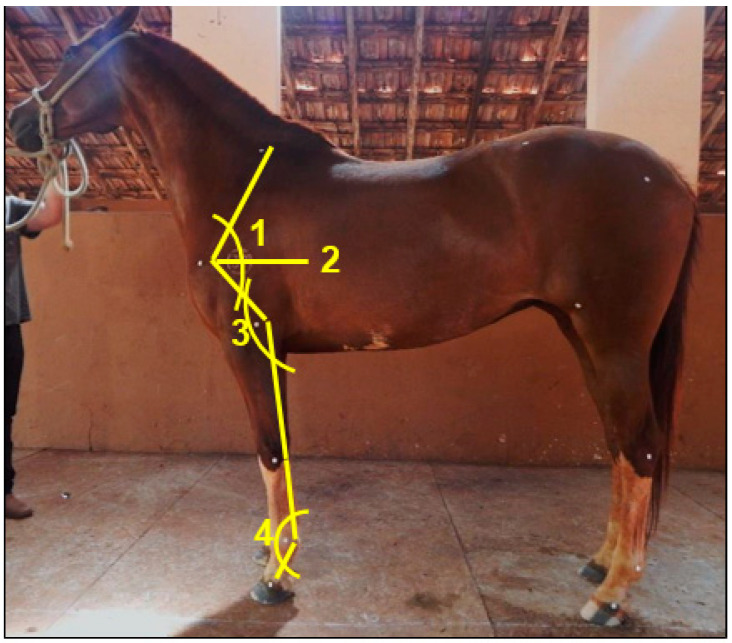
Photographic image of a female Mangalarga horse, demonstrating the joint angles measured in this study. Regarding the numbers, 1—scapulo-ground (SG), 2—scapulohumeral (SH), 3—humeroradial (HR), and 4—metacarpophalangeal (MCP).

Two evaluations were performed. The first before trimming and the second immediately after the procedure. The solar surface of the thoracic limb hooves was cleaned before data collection to remove all the solar dirt. Anatomical landmarks were used to place circular markers on the left forelimbs to ensure that the same anatomical regions were measured in all animals (Figure 1). Spherical reflective markers, 1.6 cm in diameter, were affixed to six anatomical landmarks, according to previous descriptions [18,19]. The markers were used to measure the joint angles in the left thoracic limb (Figure 2). Next, the animals were positioned in a static standing position, with the fore and hind limbs positioned perpendicular to the ground, forming a rectangular parallelogram so that the limbs were aligned and bearing weight evenly on all four limbs.

In addition to the circular markers, adhesive tape was applied to aid digital measurements of the hooves [20]. The tapes were attached to the most dorsal aspect of the hoof wall to aid the subjective adjustment of the camera to obtain the lateromedial image of the digit. The tape was also affixed to the most palmar aspect of the medial and lateral bulbs of the heel at the coronary band. During image capture, a ruler was used for calibration, and the subject and the thoracic limb under evaluation were also identified in the image. 

**Figure 3:**
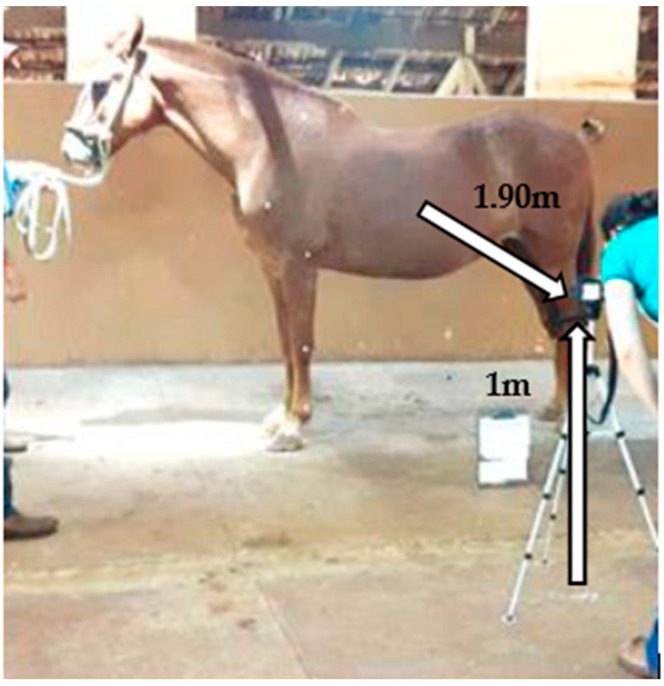
Positioning of the image capture equipment and spatial relation of the distance between the center of the camera lens in relation to the ground and in relation to the animal’s body for the photographic record of joint angles with the animal kept in a forced static quadrupedal position.

The digital camera (Nikon Coolpix L820 (ISO-200) was positioned after adjusting the static positioning of the animals. For the photographic record, the camera was positioned on a leveled tripod, with the center of the lens 1.0 m from the ground, and 1.90 m from the animals. By convention, the equipment was always kept to the side of the horse (Figure 3). 

After capturing the image of the animal in a forced static position, the camera was removed from the tripod, and each thoracic hoof was photographed.

**Figure 4:**
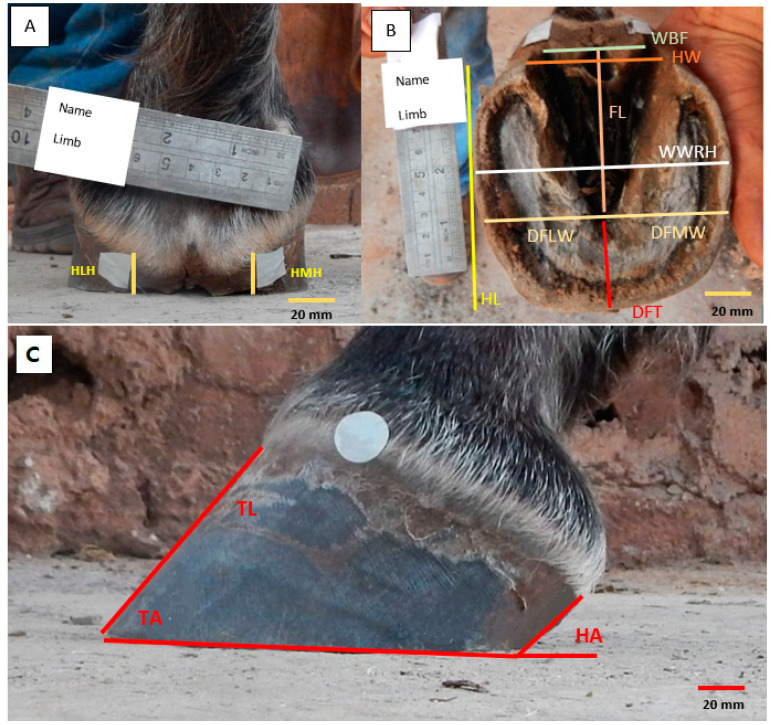
Photographic images of a Mangalarga horse’s hoof. (**A**) Palmarodorsal view; (**B**) soleus view, and (**C**) lateromedial view.

Three images were taken for each thoracic digit under evaluation (palmar view, lateromedial view, and soleus view) (Figure 4). Images of the palmarodorsal aspect of the hooves were taken first, followed by lateromedial photographs. All recordings were performed with the horses positioned squarely on a level stone surface. To record the solar view, the thoracic limbs were individually elevated, and the camera was positioned parallel to the sole.

Following the image capture, all four hooves were trimmed by a single professional, following the precepts of geometric balance previously described [1,21], keeping the horny sole intact, and the walls were kept longer (3 to 5 mm) when compared to shoe trimming and the edges were rounded [22]. The farrier had a certificate of training and previous experience of 5 years, in addition to being a member of the Farriers Association of Brazil. After trimming, the hooves were again marked with adhesive tape, and images were captured in the same way. The reflective markers were also replaced at the anatomical landmarks in case they had become detached because of the friction of the animal’s limb on the farrier’s body. It is important to highlight that the described methodology was performed continuously in the animals; thus, after all the steps described were performed in animal 1, we started animal 2, and so on.

Images were visualized using Image J software (ImageJ 1.46, U.S. National Institutes of Health, Bethesda, MA, USA). All measurements were performed by the same evaluator. Morphometric and angular measurements are listed in Table 1.

Hoof measurements and joint angles were determined in the software described above; all linear measurements were performed with the aid of the straight-line tool. The toe angles (TA), heel angle (HA), and the shoulder-ground (SG), humeroradial (HR), scapulohumeral (SH), and metacarpophalangeal (MCP) joint angles were determined using the angle tool.

Data regarding hoof measurements were submitted to a two-way analysis of variance (antimer and evaluation period), followed by Tukey’s post hoc test (*p* < 0.05); data regarding joint angles were submitted to paired t-test, and they were carried out in a using a computational statistical program (Sigmaplot, v.12.5).

Correlations between angular measurements and hoof measurements were evaluated using scatterplots and Pearson’s correlation coefficient. The statistical significance of the correlation coefficient (r) was set at *p* < 0.05. All analysis was performed using free software (Jamovi).

## 3. Results

### 3.1. Hoof Measurements

Differences were observed after trimming for the variables HL, TA, HA, and HMH. There was an increase in HL in the left hooves, an increase in TA for the left hoof and a decrease in the right hoof, and a decrease in HA and HMH values in both antimeres. The TA measurement differed between limbs before trimming (Table 2).

### 3.2. Joint Angles

There was a decrease in the MCP angle after trimming (Table 3). No further significant differences in limb angulations were noticed following trimming. 

### 3.3. Correlations between Hoof Measurements and Joint Angles

A total of 39 correlations were found. Of these, 19 were observed before trimming and 31 after the procedure (Table 4).

## 4. Discussion

**Figure 5:**
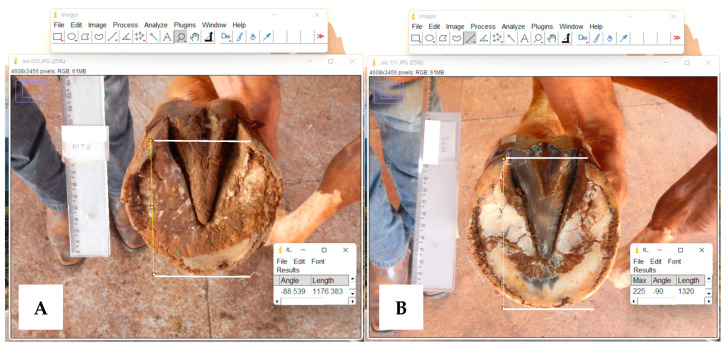
Photographic images of a Mangalarga horse’s hoof (soleus view). (**A**) Before trimming; (**B**) after trimming. The white line higher shows the support area of the palmar region of the hoof, the white line inferior shows the toe, and the cross line demonstrates the hoof length (HL) in the images. There is an evident increase in HL in (**B**) when compared to (**A**).

The animals in this study showed poor dorsal palmar balance, which significantly contributes to the long toe/heel underrun conformation. With the growth of the hoof, there was a consequent decrease in contact in the palmar region; this alteration increases the tension in the deep digital flexor tendon and can affect associated structures [1]. Trimming increases the contact surface area of the palmar region, which helps in the distribution of biomechanical forces generated during movement [23,24]; this fact explains the increase in HL in the left hoof and decreased HA in both hooves after trimming (Figure 5), characterized by greater uniformity of wall contact, increased sole contact, and increased frog and bar contact with the ground surface [25]. 

There was an increase in TA in the left hoof and a decrease in TA in the right hoof; this difference was attributed to a longer toe on the left hoof when compared to the contralateral hoof in the study animals. The farrier during trimming, using the precepts of geometric balancing [1,21], matched the TA, so there was no difference for this variable after trimming. Thus, there was an increase in TA for the right hoof and a decrease in TA for the left hoof after trimming. Previous studies have observed an increase in TA, but it is important to emphasize that these studies bring the average TA of the thoracic hooves [10] or just the values of one hoof [8]. 

Although it is reported in the literature that the ideal TA for the thoracic hooves is 45 degrees [26], this value has already been rejected in science and practice [27,28]. Variations in TA are reported [29]. However, the ideal angle for the forelimbs is between 50 and 55 degrees, which is within the same range suggested for the hindlimbs [28,30]. Only the TA before trimming for the right hoof was outside the angle considered normal in the literature; however, after trimming, the value was within the recommended values. These measurements are important, as the distribution of force on the hoof is also related to the TA, where acute angles increase the load on the heels. For example, a TA of 39 degrees results in 75% load weight on the heel bulbs compared to 57% load when the TA is “normal” at 55 degrees [31]. The increase in TA in the present study, after trimming, was 0.9 degrees, a much lower value when compared to other studies that observed an increase of 2.26 degrees [8] and 3.3 degrees [14]. We attribute this difference to the shoeing performed in the cited studies, as in the shoed horse, the presence of the shoe prevents toe wear [8].

The toe angle (TA) is defined as the angle formed by the junction of the hoof wall and its weight-bearing surface [2, 27] and is related to the hoof pastern axis (HPA) [32]. Therefore, it is an extremely important measure of dorsopalmar balance. This variable differed after trimming in our study, in similarity to the findings of two previous studies [8,10].

**Figure 6:**
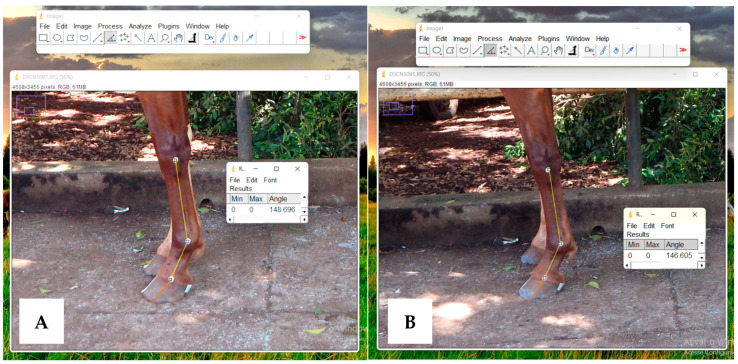
Photographic images of the forelimb of a Mangalarga mare, (**A**) before trimming and (**B**) after trimming. Demonstrating the measurement of the MCP angle in the ImageJ software, showing a decrease in the MCP angle and alignment of the hoof wall with the dorsal surface of the pastern (HPA) in (**B**).

In addition to the differences found in hoof measurements immediately after trimming, there was a significant decrease in the MCP angle. The HPA “broken backward” leads to hyperextension of the interphalangeal joint [23]. Adequate trimming aims to restore the alignment of the dorsal wall in relation to the dorsal surface of the pastern [33]. This explains the decrease in the MCP angle found in the present study (Figure 6). The compensatory mechanism for changes in hoof conformation during a regular shoeing interval is mainly located at the distal interphalangeal joint, resulting in increased load on this joint. Given the anatomy of the horses’ distal limb, the structures most affected by this increased momentum are the deep digital flexor tendon and the surrounding navicular bone [14]. In another study, this angle increased slightly after trimming [8]. It is worth mentioning that the evaluations of the aforementioned study were performed before and after trimming and shoeing, with intervals from six to eight weeks. In the shod horse, the presence of the shoe prevents toe wear. Therefore, the adaptation which occurs over time will be influenced by the shoe [8]. This fact probably explains the differences found in the present study for the MCP angle, since there are no significant differences in hoof preparation from a horse that will be shod to one that will not be shod, except that the horny sole must be left intact in barefoot animals, and the hoof walls should be kept 3 to 5 mm longer, and the edges should be rounded to a radius of at least half the wall thickness [22]. 

The ImageJ software is easy to use, free, has excellent accuracy [34], and is widely used in biological sciences [35]. It proved to be an easy and efficient program for linear and angular evaluations in our study.

## 5. Conclusions

Trimming has an immediate effect on the toe angle (dorsal angle) and heel angle (palmar angle) in Mangalarga horses, improving dorso/palmar balance and realigning the HPA. Trimming changes not only the morphometry of the hoof, but also the metacarpophalangeal angle immediately after the procedure, resulting in a decrease in this angle.

The effect of hoofing differs between shoed and barefoot animals. Barefoot animals show greater toe wear, affecting linear and angular measurements, differing from studies evaluating shoed animals [8].

Assessments carried out in a population of horses of a specific breed with the same physical activity reduce the degree of inter-individual variation and can reveal whether the trimming interval is adequate for that specific population, since each breed has particularities in the conformation of the hooves [36,37].

Longer intervals of trimming result in decreased AT by increasing palmar loading, resulting in weakening and collapse of the heels, which increases the load on the suspensory apparatus, which leads to increased susceptibility to injury [1,38], so a reduction in the trimming interval is indicated for the studied population, in order to avoid the backward break of the HPA.

## Figures and Tables

**Table 1:** Measurements were performed on the hooves and thoracic limb joint angles of Mangalarga mares (*n* = 19), acquired from digital photographs before and after trimming.

View	Abbreviation	Description
Lateral	TL	Toe Length (from the crown of the hoof to the toe)
	TA	Toe Angle (the angle formed by the hoof wall with the ground)
	HA	Heel Angle (from the angle formed by the heel with the ground)
	SG	Scapulo-Ground (Angle formed by the scapula and the ground)
	SH	Scapulohumeral (Angle formed by the scapula and humerus)
	HR	Humeroradial (Angle formed by the humerus and radius)
	MCP	Metacarpophalangeal (Angle formed by the metacarpal and the proximal phalanx)
Palmar	HMH	Height of medial heel (from a straight line starting at the hairline of the lateral heel bulb to the ground surface)
	HLH	Height of lateral heel (from a straight line starting at the hairline of the lateral heel bulb to the ground surface)
	HL	Hoof length (the distance between two parallel lines delimiting the most palmar and most dorsal support region of the hoof)
Solar	WBF	Width at the base of the frog (determined by drawing a straight line at the base of the frog)
	HW	Heel width (the distance between the lateral heel and the medial heel)
	DFLW	Distance from the apex of the frog to the lateral wall
	DFMW	Distance from the apex of the frog to the medial wall
	DFT	Distance from the apex of the frog to the toe
	WWRH	Width of the widest region of the hoof (the distance between the medial and lateral walls of the hoof in its widest region)
	FL	Frog length (from its base to its apex)

**Table 2:** Mean and standard deviation of the linear and angular variables of the thoracic hooves (left thoracic limb [LTL] and right thoracic limb [RTL]) of 19 female Mangalarga horses, obtained before (Before-trim) and after trimming (After-trim).

Variables	LTL		RTL	
	Before-Trim	After-Trim	Before-Trim	After-Trim
HL (cm)	11.6 ± 0.2 A	12.3 ± 0.2 B	11.6 ± 0.2	12.3 ± 0.2
WBF (cm)	5.3 ± 0.1	5.1 ± 0.1	5.5 ± 0.1	5.4 ± 0.1
HW (cm)	6.4 ± 0.1	6.0 ± 0.1	6.5 ± 0.1	6.3 ± 0.1
FL (cm)	8.7 ± 0.2	8.8 ± 0.2	8.6 ± 0.2	8.9 ± 0.2
FL (cm)	8.9 ± 0.1	8.4 ± 0.1	8.9 ± 0.1	8.2 ± 0.1
TA (º)	49.6 ± 0.3 aA	50.5 ± 0.3 B	51.4 ± 0.3 bA	50.8 ± 0.3 B
HA (º)	50.0 ± 1.0 A	49.9 ± 1.0 B	50.7 ± 1.0 A	50.0 ± 1.0 B
HLH (cm)	3.3 ± 0.0	2.7 ± 0.0	3.3 ± 0.0	2.7 ± 0.0
HMH (cm)	3.2 ± 0.1 A	2.7 ± 0.1 B	3.3 ± 0.1 A	2.7 ± 0.1 B
DFMW (cm)	5.7 ± 0.1	5.6 ± 0.1	5.7 ± 0.1	5.5 ± 0.1
DFLW(cm)	5.6 ± 0.1	5.6 ± 0.1	5.6 ± 0.1	5.7 ± 0.1
WWRH(cm)	12.1 ± 0.2	12.0 ± 0.2	12.1 ± 0.2	12.1 ± 0.2
DTF (cm)	4.4 ± 0.1	4.3 ± 0.1	4.3 ± 0.1	4.4 ± 0.1

Different lowercase letters on the line differ between antimeres (asymmetry), and different capital letters on the line differ between Before-trim and After-trim by Tukey’s test (*p* < 0.05). (º)—degrees; (cm)—centimeters.

**Table 3:** Mean and standard deviation of the thoracic joint angles of 19 female Mangalarga horses, obtained before (before-trim) and after trimming (after-trim).

Angles	Before-Trim	After-Trim
SG (º)	60.2 ± 4.6	58.4 ± 5.8
SH (º)	108.1 ± 4.2	107.7 ± 4.9
HR (º)	144.6 ± 6.0	144.1 ± 6.3
MCP (º)	146.3 ± 6.0 a	143.5 ± 6.3 b

Different lowercase letters on the line differ between times by Tukey’s test (*p* < 0.05). (º)—degrees.

**Table 4:** Correlations between the variables measured in the thoracic limb hooves and forelimb joint angles before trimming (before-trim) and after trimming (after-trim).

Variables		Before-Trim		After-Trim	
		*r*	*p*	*r*	*p*
**HMH**	HLH	0.534 *	0.019	0.647 **	0.003
**HL**	HMH	−0.499 *	0.030	−0.144	0.557
**DFT**	HL	0.571 *	0.011	0.808 ***	<0.001
**WWRH**	HMH	−0.544 *	0.016	−0.470 *	0.042
**HW**	HL	0.467 *	0.044	0.561 *	0.013
**HW**	WWRH	0.503 *	0.028	0.626 **	0.004
**WBF**	HL	0.503 *	0.028	0.406	0.085
**WBF**	WWRH	0.584 **	0.009	0.522 *	0.022
**WBF**	HW	0.942 ***	<0.001	0.927 ***	<0.001
**DFLW**	WWRH	0.526 *	0.021	0.736 ***	<0.001
**DFMW**	WWRH	0.544 *	0.016	0.843 ***	<0.001
**DFMW**	DFLW	0.697 ***	<0.001	0.811 ***	<0.001
**SH**	FL	−0.457 *	0.049	−0.202	0.407
**SH**	DFLW	0.690 **	0.001	−0.245	0.312
**HA**	HLH	0.493 *	0.032	0.405	0.085
**TL**	FL	0.506 *	0.027	0.592 **	0.008
**TL**	SG	−0.553 *	0.014	−0.363	0.127
**TA**	HLH	0.472 *	0.041	0.094	0.702
**HMH**	HA	0.253	0.295	0.617 **	0.005
**HL**	TL	0.424	0.071	0.609 **	0.006
**FL**	HL	0.233	0.337	0.835 ***	<0.001
**DFT**	TL	0.392	0.097	0.512 *	0.025
**DFT**	FL	0.133	0.588	0.613 **	0.005
**DFLW**	DFT	−0.339	0.155	0.468 *	0.043
**DFMW**	HL	0.266	0.271	0.489 *	0.034
**DFLW**	DFT	−0.263	0.277	0.644 **	0.003
**WWRH**	DFT	0.051	0.837	0.651 **	0.003
**WWRH**	HL	0.362	0.128	0.703 ***	<0.001
**WWRH**	FL	0.319	0.183	0.651 **	0.003
**HW**	FL	0.187	0.443	0.745 ***	<0.001
**HW**	DFT	0.384	0.105	0.506 *	0.027
**HW**	DFMW	0.079	0.747	0.511 *	0.026
**WBF**	FL	0.284	0.238	0.614 **	0.005
**WBF**	DFMW	0.142	0.563	0.475 *	0.040
**SH**	SG	0.069	0.780	0.488 *	0.034
**HR**	SG	−0.425	0.070	−0.071	0.774
**MCP**	SG	0.364	0.125	−0.445	0.064
**MCP**	SH	0.199	0.414	−0.204	0.416
**HR**	SH	0.547 *	0.015	0.669 **	0.002

* *p* < 0.05, ** *p* < 0.01, *** *p* < 0.001.

## Data Availability

The raw/processed data required to reproduce these findings are available from the corresponding author upon reasonable request.
